# Measuring The Enduring Imprint Of Structural Racism On American Neighborhoods

**DOI:** 10.1377/hlthaff.2023.00659

**Published:** 2023-10

**Authors:** Zachary Dyer, Matthew J. Alcusky, Sandro Galea, Arlene Ash

**Affiliations:** University of Massachusetts Chan Medical School, Worcester, Massachusetts; University of Massachusetts; Boston University, Boston, Massachusetts; University of Massachusetts

## Abstract

A long history of discriminatory policies in the United States has created disparities in neighborhood resources that shape ethnoracial health inequities today. To quantify these differences, we organized publicly available data on forty-two variables at the census tract level within nine domains affected by structural racism: built environment, criminal justice, education, employment, housing, income and poverty, social cohesion, transportation, and wealth. Using data from multiple sources at several levels of geography, we developed scores in each domain, as well as a summary score that we call the Structural Racism Effect Index. We examined correlations with life expectancy and other measures of health for this index and other commonly used area-based indices. The Structural Racism Effect Index was more strongly associated with each health outcome than were the other indices. Its domain and summary scores can be used to describe differences in social risk factors, and they provide powerful new tools to guide policies and investments to advance health equity.

## Introduction

The COVID-19 pandemic brought into sharp relief both faults in the health care system that impede people with fewer resources from maintaining health and the many ways in which social structures reinforce disparities in the resources that support healthy lives. Black, Latine, and Indigenous residents were more likely than White residents to be exposed to and die from COVID-19 for many reasons: They were more likely to be essential workers and less able to take time off or to work at home, more likely to take public transportation, less likely to be able to quarantine at home, more likely to have comorbid conditions, and less likely to have ongoing help in managing their comorbidities.^1^ People were becoming sick and dying at different rates because of deeply embedded structural differences in their neighborhoods.^2^

Social determinants of health, or the conditions in which people grow, live, learn, and work, are primary drivers of most health outcomes.^3^ This is just as true for COVID-19 as it is, for example, for stroke, cardiovascular disease, and cancer.^4^ Further, health disparities across ethnoracial groups in the US persist despite awareness of and long-term efforts to address them. Compared with White populations, Black, Latine, and Indigenous populations have lower life expectancy, poorer health outcomes, and less access to health care and health-supporting resources.^5^ Underlying these disparities are centuries of policies that harm populations of color, starting with American colonialism and slavery and extending to unequal access to Medicaid expansion and beyond, leading to current deficits in the availability of health-supporting resources in the places where they disproportionately live.^4,6^

Geographic resource deprivation that closely aligns with where Black and Indigenous populations live today is the result, most specifically, of segregation and, more broadly, of structural racism, which is defined here, following Zinzi Bailey and colleagues, as “the totality of ways in which societies foster racial discrimination, through mutually reinforcing inequitable systems…that in turn reinforce discriminatory beliefs, values, and distribution of resources, which together affect the risk of adverse health outcomes.”^4^ These “mutually reinforcing inequitable systems” include housing, employment, criminal justice, and more—social factors that strongly influence health. Despite structural racism being upstream to inequities in social determinants of health and in health, its contributions to inequity are infrequently quantified or considered.^7,8^

Some measures of structural racism exist, but they have important limitations. For example, existing measures are often limited to a single domain, such as housing, criminal justice, employment, or education,^9^ and single-dimension approaches fail to capture the importance of the mutually reinforcing inequitable systems highlighted by Bailey and colleagues.^4,10–12^ The few efforts to integrate multiple domains have looked only at larger geographical units: counties, states, or large statistical areas.^11^ These approaches miss much of the nuance of neighborhood-level variation and the compounding effects of multiple policies that leave some cities with a greater than fifteen-year gap in life expectancy between majority-Black and majority-White neighborhoods within their boundaries.

There are more widely used indices to measure neighborhood-level social risks, but these, too, do not fully capture the upstream aspects of discriminatory policies. These measures are variously referred to as measures of stress, deprivation, vulnerability, or (lack of) opportunity, and they typically emerge from composite scores derived from publicly available data. The Neighborhood Atlas Area Deprivation Index (ADI),^13,14^ the Centers for Disease Control and Prevention’s (CDC’s) Social Vulnerability Index (SVI),^15^ the Social Deprivation Index (SDI),16 and the Child Opportunity Index (COI)^17^ are frequently cited. Their uses include public health planning, cost prediction, and risk-adjusting payment models to compensate providers that care for people from under-resourced neighborhoods.^18^ The literature is rich on their utility, and as health care increasingly seeks to address health-related social needs, their importance is increasing.^19,20^

Existing measures, however, do not measure resource availability at the community level as clearly or comprehensively as would be useful to policy makers. Some were developed for specific applications (for example, the SVI to identify areas needing greater attention during natural disasters and the COI to understand child health).^15,17^ They might not consistently predict outcomes across urban and rural areas, or they may fail to capture important differences across population densities.^21,22^ No measure specifically considers the legacy of structural racism in its construction, and the structural racism literature has identified concerns with some measures. For example, some measures confuse the effects of racism with race itself by allowing the presence of Black or Latine populations to serve as a measure of disadvantage.^10,12^ With the exception of the COI, most measures do not include data from a range of fields and disciplines, potentially missing the multidimensional nature of disadvantage.

We have addressed several shortcomings of existing indices by creating multidimensional census tract–level measures and a summary score that individually and collectively identify contemporaneous differences in the resources available to communities, as shaped by a long history of racially inequitable policies. In this study we compared our index with other leading indices, hypothesizing that ours, which was explicitly constructed using a structural racism framework, would better predict health inequities. Our work can help health care and other organizations identify and address structurally embedded inequities in access to health care, housing, education, and other resources.

## Study Data And Methods

### Domain Identification

We constructed the Structural Racism Effect Index in multiple steps, beginning with domain identification. We reviewed the literature to identify relevant domains of the effects of structural racism. We collapsed the conceptually distinct domains of “income” and “poverty” because most poverty measures are based on income, leaving nine domains: built environment, criminal justice, education, employment, housing, income and poverty, social cohesion, transportation, and wealth. Supplement 1 in the online appendix lists the literature used to identify these domains.^23^

### Variable Collation And Selection

We cast a wide net in seeking variables for potential inclusion in the index. First, we systematically reviewed every variable available from the Census Bureau’s American Community Survey 2015–19 five-year estimates. We included any variable that both nominally fit one of our identified domains and was not the complement of a binary variable already included. Next, we queried the federal government’s Data.gov database catalog, using the names of original or final domains. Finally, we reviewed contemporary literature on social determinants of health and structural racism to identify other data sets used to describe the neighborhood conditions that influence health and well-being by querying PubMed with the terms “census tract” and either “social determinants” or a domain name (for example, “census tract AND housing”).

We constructed domain scores (and the Structural Racism Effect Index) at the census tract level, balancing availability of data with closest approximation to neighborhood.^24,25^ For inclusion in the index, data must have been available across the entire US, preferably measured at the census tract level. For some variables, data were only available for larger geographical units. Because geographical units larger than census tracts convey less information about neighborhood differences, we sought to include them only when that level of granularity was policy appropriate. For example, as county governments frequently maintain jails, we included incarceration data at the county level, allowing census tracts to “inherit” characteristics from counties that contained them.

Although we examined many variables, we sought fewer variables for the final index, prioritizing simplicity for end users. Exhibit 1 shows all of the variables included in the final index. Supplement 2 in the appendix provides data sources used for variables and additional details on variable selection and transformation, and it describes how differing geographies were handled.^23^

### Index Construction

We oriented all selected variables in the same direction, so that higher values always indicate disadvantage: fewer resources, higher stress, and poorer outcomes. We recoded some variables with many very low or zero values, using the cube root transformation (noted in exhibit 1).

We standardized each variable, summed standardized values in each domain, and restandardized the sum. We calculated the Structural Racism Effect Index by adding the nine domain scores and standardizing once more; thus, each domain score and the Structural Racism Effect Index have a mean of 0 and a standard deviation of 1. All standardization calculations were weighted by census tract population.

For each domain score and the Structural Racism Effect Index, negative scores indicate areas that were richer in resources, and positive scores indicate areas that were poorer in resources. We calculated the Structural Racism Effect Index for every US census tract with at least 100 households with the exception of those with missing data, either for all variables in any domain or for more than nine variables total. We used geo-imputation to account for most other missing data by taking the median value of at least two contiguous census tracts and assigning the national average to values that could not be geo-imputed. Supplement 3 in the appendix quantifies excluded tracts and missing data issues.^23^ Less than 3 percent of data used in the Structural Racism Effect Index were imputed.

#### Index Validation Data Sources

We used census tract–level American Community Survey data on ethnoracial groups for several analyses. For this study, we defined “Black” as anyone whose single race or race in combination with others was Black; “Indigenous” as anyone whose single race or race in combination with others was Indigenous or Native American but who did not identify as Black; “Latine” as anyone whose ethnicity was Hispanic or Latine but who did not identify as Black, Indigenous, or Native American; and a combined category, “people of color,” as anyone who fit into one of these three categories. We did not include those who identified as Asian in this definition because the racism faced by Asian people within the US is distinct from the historical structural resource deprivation as a legacy of slavery and American colonialism that we aimed to measure. This is not intended to minimize the racism the Asian community has faced and continues to face.

We compared the Structural Racism Effect Index with the ADI, SVI, SDI, and COI. Using 2015–19 American Community Survey data, we replicated the ADI national percentile score at the census tract level, using methods described in the literature.^13,26,27^ We downloaded the 2019 SVI total scores for census tracts from the CDC website. We downloaded the 2019 SDI total scores for census tracts from the Robert Graham Center. Finally, we downloaded the 2015 COI scores for census tracts from diversitydatakids.org. More information about these other indices is in supplement 2 in the appendix.^23^

Average life expectancy was available for 88.8 percent of US census tracts through the National Center for Health Statistics’ U.S. Small-area Life Expectancy Estimates Project, using 2010–15 data.^28^ These data are modeled using decennial census and American Community Survey population estimates and two social determinants of health measures: quartile of median family income and percentage of the population with a bachelor’s degree.^28^

This article’s health measures (mental health “not good” for fourteen or more days during the past thirty days; physical health “not good” for fourteen or more days; and self-reported diagnoses of diabetes, high blood pressure, and asthma) came from the CDC PLACES project, which derives small-area estimates using multilevel modeling.^29,30^

#### Index Validation

We first examined face validity by categorizing census tracts into deciles of Structural Racism Effect Index scores and domain scores. We took population-weighted averages by decile of life expectancy, percentage of the census tract reporting diabetes, and percentage of the population that were people of color (defined as explained above). We expected that, on average, life expectancy would decrease as deciles of the Structural Racism Effect Index increased, whereas both diabetes prevalence and percentage of the population that were people of color would increase.

We examined the performance of five indices (the Structural Racism Effect Index, ADI, SVI, SDI, and COI) in several ways. Primarily, we examined correlations with health outcomes that reflect long-lasting resource deprivation. We compared R^2^ values from univariate linear regression models predicting average census tract values for each of the five indices and six health outcomes (life expectancy, poor mental health, poor physical health, diabetes, high blood pressure, and asthma). We compared R^2^ values among all census tracts and separately for tracts stratified by degree of rurality and by ethnoracial composition. We hypothesized that our index, explicitly constructed using a structural racism framework, would predict poor health outcomes better than existing indices, especially among urban census tracts and among those with mixed ethnoracial makeup.

We categorized census tracts as metropolitan, micropolitan, small town, or rural on the basis of the rural-urban commuting area codes used by the Census Bureau. In addition, we classified census tracts as having a high proportion of people of color (defined as explained above) when the combined proportion of Black, Latine, and Indigenous people exceeded 50 percent of the population, as a mixed area when that proportion was between 10 percent and 50 percent, and as having a low proportion of people of color when that proportion was less than 10 percent. This classifies roughly one-quarter of all census tracts in each of the high and low categories, and half in the mixed category.

### Limitations

This work had several limitations, with the lack of availability of nationally comprehensive and credible data being the most significant. Data on voting, eviction, and criminal justice were hard to find, compare, and integrate. Only some states make such data readily available. Limited criminal justice data are a particular challenge, as there are substantial ethnoracial disparities that affect health within this system. In addition, no voting data were available nationwide for inclusion in the final social cohesion domain. The Massachusetts Institute of Technology Election Data and Science Lab,^31^ the Princeton Eviction Lab,^32^ the Vera Institute,^33^ and many others are working to improve data quality and availability in these important areas.

Our health-related variables were small-area estimates and may have relied on modeling using demographic data. For example, census tract–level life expectancy is estimated by the U.S. Small-area Life Expectancy Estimates Project from a model using seven sociodemographic variables, including one measure of income and one of educational attainment. This commonality may falsely increase correlations between indices and life expectancy.

Another concern relates to the need to choose weights for any summary score. For simplicity, we weighted all variables equally within each domain and all domains equally in constructing the Structural Racism Effect Index. Although simple to understand and easy to replicate, this was arbitrary and made no attempt to account for differences in the relative importance for health, the reliability of individual variables or domain scores, or correlations among variables and domain scores. There is no consensus on how to choose weights, and all weighting methods appear to be poorly understood by end users.^25,34,35^

## Study Results

We calculated Structural Racism Effect Index scores for 71,917 census tracts across fifty states; Washington, D.C.; and Puerto Rico, whose populations total an estimated 323,733,294 people, or more than 97 percent of the US population. The mean index score was 0.08, with a standard deviation of 1.04 (the population-weighted mean was 0 with standard deviation equal to 1). The minimum score was −4.28, and the maximum score was 5.02; 90 percent of scores fell between −1.57 and 1.69 (data not shown).

We divided census tracts into deciles of three key outcomes (life expectancy, diabetes prevalence, and percentage identifying as people of color), indicating their full, national, neighborhood-level range. We then examined deciles of the Structural Racism Effect Index and domain scores to see how much of that variation was captured by each. Deciles of the Structural Racism Effect Index and domain scores are monotonically related to each outcome. For census tracts in the most-resourced Structural Racism Effect Index decile (decile 1), average area life expectancy was 82.6 years, mean diabetes prevalence was 7.6 percent, and the average proportion of the population identifying as people of color was 14.7 percent. In marked contrast, the analogous numbers in the least-resourced decile (decile 10) were 73.0 years, 17.5 percent, and 69.1 percent (exhibit 2). For every domain, the decrease in life expectancy from decile 1 to decile 10 was greater than four years. See appendix supplement 4 for a decile-level visualization.^23^

The Structural Racism Effect Index was most strongly correlated with life expectancy among all comparator indices studied. Nationally, and within subgroups of census tracts (such as those in rural areas or areas with mixed ethnoracial makeup), the Structural Racism Effect Index consistently achieved the highest R^2^ values of any index (exhibit 3).

The Structural Racism Effect Index was also more consistently associated with other health outcomes (exhibit 4), particularly with poor mental and physical health. The Structural Racism Effect Index explained far more of the variation in health status, with R^2^ values above 0.7 compared with around 0.5 for all indices except the COI, which had values above 0.6.

Supplement 5 in the appendix includes a map visualizing Structural Racism Effect Index scores across the country.^23^

## Discussion

Significant investments in health care have been made to address health-related social needs—for example, with outreach workers, housing vouchers, and after-school programs. However, equity gaps persist, as many current policies reinforce, or do nothing to address, structurally embedded inequities in access to health care, housing, education, and other resources.

Using publicly available data on social determinants of health, we constructed scores for US census tracts in domains known to carry the major imprint of structural racism, and we created from them the Structural Racism Effect Index. This summary index correlated more strongly with life expectancy and other health measures than several widely used area-level measures of social determinants of health.

Several area-level indices of social determinants of health risk exist; they vary by the number, nature, and sources of included variables; geographic granularity; and how individual variables are weighted.^35^ Most are available as single-number summaries only, instead of being transparently constructed from distinct domains. We identified domains that could not be missing when trying to capture the many ways in which structural racism influences neighborhood resources. We interrogated multiple data sources to ensure that these domains were as informative as possible.

So that others can make use of this work, we have made the domain scores, Structural Racism Effect Index scores and percentiles, and other details about the index freely available online.^36^ A neighborhood’s multidimensional profile can help inform strategies to reduce inequity.

Neither addressing nor measuring structural racism is straightforward specifically because it is multidimensional in nature. Any neighborhood measure of social determinants of health will capture some of these effects, but ours is the first to include all domains central to the negative synergies that perpetuate racial inequity, including criminal justice and social cohesion. We purposely sought variables and domains that may be absent from, or receive too little weight in, existing measures. For example, because of the ADI’s reliance on home value,^22^ its correlation with the Structural Racism Effect Index wealth domain is 91 percent. However, the ADI is less correlated with our social cohesion, criminal justice, and education domains (38 percent, 22 percent, and 62 percent; data not shown).

Our findings are consistent with the hypothesis that centuries of policies that explicitly and implicitly maintained segregation and limited access to resources drive current inequities in all nine domains. Cycles of disinvestment in areas with concentrated Black, Indigenous, and eventually Latine residents began with policy choices—from colonization to slavery to segregation to allowing racial terror to withholding resources for wealth-accumulating homeownership and beyond. Thus, the present-day contribution of disinvested neighborhoods to health inequity was largely intentional and certainly preventable. Policies to mitigate the harm of structural racism must likewise be intentional. This work provides insights for individuals and organizations working toward racial justice. It could be used to help guide local investments to equitably develop green space or to inform hospital community benefit programs, local voter participation efforts, a university’s scholarship commitments, or other initiatives that seek to share power or redistribute resources to counter structural racism’s effects.

There are many potential uses of the Structural Racism Effect Index in research. An individual’s ethnoracial group is often used in research and evaluation as a proxy for their experiences of racism (interpersonal and structural) but is rarely properly identified in this way.^37^ The Structural Racism Effect Index score of an individual’s neighborhood used in this context would represent a more conceptually accurate measure of exposure to structural racism or provide important context in addition to their ethnoracial identity. For example, accountable care programs providing housing vouchers or food-as-medicine supports can test whether those programs have greater or lesser impacts for people who live in areas with higher effects of structural racism and tailor their programs accordingly.

The Structural Racism Effect Index can also be used by communities for advocacy and strategic planning; for telling their stories; and in campaigns to reverse the effects of past unjust, racially biased policies and address root causes of inequity.

An important feature of the Structural Racism Effect Index is that it is almost entirely race-neutral (the social cohesion domain includes information about racial segregation, but no direct measure of ethnoracial composition). We adopted this approach for several reasons. First, although many modern so-called race-neutral policies are racist in that they disproportionately harm Black and Indigenous residents, their harms are not limited to those groups.^38^ Second, ethnoracial categorization is inherently problematic and ignores significant heterogeneity and starkly different realities for communities within commonly used ethnoracial categories.^39^ Third, in the current US political climate, there is a growing need to address ethnoracial disparities in a way that does not rely explicitly on race. The June 2023 Supreme Court decision Students for Fair Admissions, Inc. v. President and Fellows of Harvard College, for example, compels universities to largely abandon race-informed admissions but lets stand the use of geography in the admissions process.^40^ Thus, the Structural Racism Effect Index could provide a legally acceptable antiracist approach to increasing admissions of underrepresented students by increasing admissions for people who are disproportionately, but not exclusively, “of color.” Further, such an approach might address the concern that affirmative action has historically done little to benefit Black, Latine, and Indigenous students who are low income or descendants of enslaved Americans.^40^

Some areas, such as parts of Appalachia, have few people of color (as we have defined them) but high Structural Racism Effect Index scores. Regardless of the extent to which the structural deficits they experience flow from the similar cycles of discriminatory disinvestment that we sought to capture, the Structural Racism Effect Index quantifies those deficits in a race-neutral way. The ethnoracial demographics most relevant for understanding and addressing existing inequities in neighborhood-level resources may differ by region or state.

There is room to sharpen the Structural Racism Effect Index, particularly in areas where structural racism is acutely present and existing data are poor.

## Conclusion

We hope that others will build on this work to develop refined indices tailored for their needs. State governments can use our index “out of the box” or can refine individual domains with more granular data—for example, on criminal justice, voting, or wealth. Should an end user wish to examine influences on a particular outcome, the Structural Racism Effect Index domains could be weighted to most strongly predict that outcome. We intend to maintain the index as a public resource and hope to publish improvements developed through collaborations with experts in several domain fields to best identify the effects of structural racism that are both measurable and critical to community realities of inequity.

The multidimensional nature of structural racism makes redressing its harms difficult. We hope that this work helps identify the places where strategies that can dismantle ingrained and self-reinforcing patterns of disinvestment can be applied to achieve a more equitable future.

## Supplementary Material

Appendix

## Figures and Tables

**Exhibit 1.** Domains and variables included in the Structural Racism Effect Index

Domain	American Community Survey Variables	Other federal data	Other social determinant data
**Built Environment**	● Building vacancy rate ● Mobile homes ● No internet access	● Cancer risk(a)(1) ● Low food access for SNAP recipients(2)	*Not applicable*
**Criminal Justice**	*Not applicable*	● Pretrial jail rate (county)(a)(3) ● Total jail rate (county)(a)(3) ● Law enforcement personnel per capita (municipality)(a)(4)	*Not applicable*
**Education**	● Bachelor’s degree or higher ● High school diploma	● Per pupil spending (school district)(5)	*Not applicable*
**Employment**	● Unemployed ● White-collar occupation	● Retail job availability(6)	*Not applicable*
**Housing**	● Housing units without telephone ● Housing units without plumbing(a) ● Crowding ● Group quarters	● Foreclosure risk(7)	● Eviction rate (a)(9)
**Income & Poverty**	● Below 100% poverty line ● Below 200% poverty line ● Public assistance ● Family income ● Per capita income	● Supplemental poverty measure(8)	*Not applicable*
**Social Cohesion**	● Changed address in last year ● Single-parent households ● Income gap(a)	*Not applicable*	● Residential Segregation(10)
**Transportation**	● Carpooled to work ● No access to a motor vehicle ● Took public transit to work ● Biked to work ● Walked to work	● Transportation cost burden, median income family(6)	*Not applicable*
**Wealth**	● Aggregate home value ● Median real estate taxes paid ● Median home value ● Median gross rent ● Median monthly mortgage ● Owner-occupied homes	*Not applicable*	*Not applicable*

**Sources**: All variables in column 2 come from the Census Bureau, American Community Survey, 2015–19 5-year estimates. For others, see specified table notes below, referring to note numbers in a list of sources in supplement 2 in the appendix (see note 23 in text). **Notes**: All data are at the census tract level unless otherwise indicated. SNAP is Supplemental Nutrition Assistance Program. FPL is federal poverty level.

a Variable transformed by cube root.

b Environmental Protection Agency, Air Toxics Screening Assessment, 2018 (note 18).

c Not applicable.

d Department of Agriculture, Food Access Research Atlas, 2021 (note 19).

e Department of Justice, Bureau of Justice Statistics Annual Survey of Jails and Census of Jails, via the Vera Institute, 2016 (note 20).

f Federal Bureau of Investigation, Uniform Crime Reporting Program, law enforcement employee data, 2019 (note 21).

g Census Bureau, Annual Survey of School System Finances, 2019 (note 22).

h Department of Housing and Urban Development and Department of Transportation, Location Affordability Index, 2019 (note 23).

i Department of Housing and Urban Development, Office of Policy Development and Research, Neighborhood Stabilization Program, 2010 (note 24).

j Princeton Eviction Lab National Database, 2016 (note 26).

k Census Bureau, Supplemental Poverty Measure, 2018 (note 25).

l National Cancer Institute, Healthcare Delivery Research Program, Social Determinants of Health by Census Tract, 2012 (note 27).

**Exhibit 2.** Mean life expectancy (2010–15), diabetes prevalence (2020), and ethnoracial makeup (2015–19) by decile of Structural Racism Effect Index (SREI)

	Life Expectancy (years)			% Reporting Diabetes			% People of Color		

	Decile 1	Decile 10	Mean increase per decile (SD)	Decile 1	Decile 10	Mean increase per decile (SD)	Decile 1	Decile 10	Mean increase per decile (SD)
**National distribution**	85.0	70.9	−1.6 (0.8)	5.9%	19.1%	1.5% (1.1%)	2.8%	92.8%	10% (6.5%)

SREI	82.6	73.0	−1.1 (0.4)	7.6%	17.5%	1.1% (0.8%)	14.7%	69.1%	6% (4.5%)

Wealth	82.6	72.9	−1.1 (0.5)	8.1%	16.4%	0.9% (0.5%)	19.6%	59.2%	4.4% (5.6%)
Income & Poverty	82.5	73.9	−1 (0.3)	9.4%	13.6%	0.5% (0.4%)	13.7%	74.9%	6.8% (5.4%)
Social Cohesion	81.4	73.3	−0.9 (0.6)	7.8%	15.3%	0.8% (0.2%)	10.4%	75.3%	7.2% (4.3%)
Employment	81.9	73.8	−0.9 (0.4)	7.9%	16.2%	0.9% (0.6%)	22.0%	63.7%	4.6% (4.9%)
Built Environment	81.7	73.9	−0.9 (0.3)	9.0%	14.4%	0.6% (0.3%)	15.9%	51.1%	3.9% (1.4%)
Housing	81.4	74.5	−0.8 (0.3)	7.7%	16.7%	1% (0.6%)	14.4%	62.6%	5.4% (2.4%)
Education	82.2	75.5	−0.7 (0.3)	9.0%	16.1%	0.8% (0.9%)	17.7%	68.4%	5.6% (6.4%)
Transportation	80.0	75.3	−0.5 (0.4)	9.1%	15.1%	0.7% (0.5%)	37.9%	42.7%	0.5% (3.3%)
Criminal Justice	79.9	75.7	−0.5 (0.6)	8.2%	15.9%	0.9% (0.5%)	22.7%	38.0%	1.7% (3.6%)

**Sources:** Mean life expectancy data are from National Center for Health Statistics. U.S. Small-area Life Expectancy Estimates Project (see note 28 in text), data for 2010–15. Diabetes prevalence data are from the Centers for Disease Control and Prevention’s PLACES Project (see note 29 in text), data for 2020. Ethnoracial makeup data are from the Census Bureau, American Community Survey, 2015–19 5-year estimates.

**Notes:** For each index, the population is divided into deciles of increasing risk; only deciles 1 and 10 are shown. Three outcomes are shown: mean life expectancy; percent reporting diabetes; and percent identifying as people of color, defined as those who identify as Black, Latine, or Indigenous. The top row of data is the mean value of each outcome by decile. The SREI was calculated by the authors, using data from the sources listed in the exhibit 1 notes, which correspond to a full list of sources in supplement 2 in the appendix (see note 23 in text).

**Exhibit 3.** Explanatory power of the Structural Racism Effect Index (SREI) and other indices for predicting census tract–level variation in life expectancy, 2010–15

	# tracts	SVI (R-squared)	SDI (R-squared)	ADI (R-squared)	COI (R-squared)	SREI (R-squared)
**Overall**	65,661	.28	.33	.43	.45	.50
**Metropolitan**	51,562	.28	.32	.47	.47	.52
**Micropolitan**	6,044	.27	.34	.32	.38	.40
**Small town**	2,998	.23	.32	.30	.29	.34
**Rural**	2,705	.17	.22	.21	.24	.30
**Low POC area (<10%)**	16,103	.30	.34	.38	.41	.45
**Mixed area (10–50%)**	32,368	.25	.29	.45	.43	.48
**High POC area (>50%)**	17,190	.12	.19	.37	.39	.43

**Sources:** Mean life expectancy (available for 88.8 percent of census tracts) was calculated from National Center for Health Statistics. U.S. Small-area Life Expectancy Estimates Project (see note 28 in text), data for 2010–15. The Structural Racism Effect Index (SREI) was calculated by the authors, using data from the sources listed in the exhibit 1 notes, which correspond to a full list of sources in supplement 2 in the appendix (see note 23 in text). The Area Deprivation Index (ADI) was calculated at the census tract level by the authors using Census Bureau American Community Survey 2015–19 5-year estimates. The Social Vulnerability Index (SVI) is from Centers for Disease Control and Prevention, Agency for Toxic Substances and Disease Registry. CDC/ATSDR Social Vulnerability Index [Internet]. Atlanta (GA): CDC; [last reviewed 2023 Jul 12; cited 2023 Aug 26]. Available from: https://www.atsdr.cdc.gov/placeandhealth/svi/ (data for 2019). The Social Deprivation Index (SDI) is from Robert Graham Center. Social Deprivation Index (see note 16 in text), data for 2019. The Child Opportunity Index (COI) is from diversitydatakids.org. Child Opportunity Index (COI) [Internet]. Waltham (MA): Brandeis University; c 2023 [cited 2023 Aug 25]. Available from: https://www.diversitydatakids.org/child-opportunity-index (data for 2015).

**Notes:** The unit of analysis is the census tract. Census tracts are classified as having a high proportion of people of color (“high POC area”) when the combined proportion of Black, Latine, and Indigenous people exceeded 50 percent of the population; as a mixed area when that proportion was between 10 percent and 50 percent; and as having a low proportion of people of color (“low POC area”) when that proportion was less than 10 percent.

**Exhibit 4:** Explanatory power of the Structural Racism Effect Index (SREI) and other indices for predicting census tract–level variation in disease prevalence, 2020

	Prevalence	SVI (R-squared)	SDI (R-squared)	ADI (R-squared)	COI (R-squared)	SREI (R-squared)
**Hypertension**	31%	.15	.09	.39	.27	.35
**Diabetes**	11%	.44	.35	.42	.54	.57
**Asthma**	10%	.34	.37	.37	.54	.48
**Poor mental health**	14%	.51	.58	.53	.67	.73
**Poor physical health**	13%	.55	.48	.53	.65	.71

**Sources:** All health outcomes are from the Centers for Disease Control and Prevention’s (CDC’s) PLACES Project (see note 29 in text), data for 2020. Self-reported disease prevalence data are from the CDC Behavioral Risk Factor Surveillance System, and small-area estimates are from the CDC’s PLACES Project. The SREI was calculated by the authors, using data from the sources listed in the exhibit 1 notes, which correspond to a full list of sources in supplement 2 in the appendix (see note 23 in text). The Area Deprivation Index (ADI) was calculated at the census tract level by the authors using Census Bureau American Community Survey 2015–19 5-year estimates. The Social Vulnerability Index (SVI) is from Centers for Disease Control and Prevention, Agency for Toxic Substances and Disease Registry. CDC/ATSDR Social Vulnerability Index [Internet]. Atlanta (GA): CDC; [last reviewed 2023 Jul 12; cited 2023 Aug 26]. Available from: https://www.atsdr.cdc.gov/placeandhealth/svi/. The Social Deprivation Index (SDI) is from Robert Graham Center. Social Deprivation Index (see note 16 in text), data for 2019. The Child Opportunity Index (COI) is from diversitydatakids.org. Child Opportunity Index (COI) [Internet]. Waltham (MA): Brandeis University; c 2023 [cited 2023 Aug 25]. Available from: https://www.diversitydatakids.org/child-opportunity-index. **Note:** The unit of analysis is the census tract.
