# Supplementary material for: Measuring The Enduring Imprint Of Structural Racism On American Neighborhoods
Source: Health Aff (Millwood). Author manuscript; Available in PMC 2024 Jan 23. (PMC10804769; doi:10.1377/hlthaff.2023.00659)
Supplement: Appendix [file NIHMS1953011-supplement-Appendix.pdf]

APPENDIX

Supplement 1

We reviewed the literature to identify all relevant domains of data that have been used to measure structural racism. The literature used is provided below. The final domains are: built environment,(1,2) criminal justice,(3–6) education,(3,4,7) employment,(4,7–9) housing,(5,10) income & poverty,(3,4,9,11) social cohesion,(6,8,12–17) transportation,(10) and wealth(8,17).

1. Tackett KJ, Jenkins F, Morrell DS, McShane DB, Burkhart CN. Structural racism and its influence on the severity of atopic dermatitis in African American children. Pediatr Dermatol. 2020 Jan;37(1):142–6.
2. Scott J, Danos D, Collins R, Simonsen N, Leonardi C, Scribner R, et al. Structural racism in the built environment: Segregation and the overconcentration of alcohol outlets. Health Place. 2020 Jul;64:102385.
3. Wallace M, Crear-Perry J, Richardson L, Tarver M, Theall K. Separate and unequal: Structural racism and infant mortality in the US. Health Place. 2017 May;45:140–4.
4. Dougherty GB, Golden SH, Gross AL, Colantuoni E, Dean LT. Measuring Structural Racism and Its Association With BMI. Am J Prev Med. 2020 Oct;59(4):530–7.
5. Alson JG, Robinson WR, Pittman L, Doll KM. Incorporating Measures of Structural Racism into Population Studies of Reproductive Health in the United States: A Narrative Review. Health Equity. 2021 Dec;5(1):49–58.
6. Chambers BD, Erausquin JT, Tanner AE, Nichols TR, Brown-Jeffy S. Testing the Association Between Traditional and Novel Indicators of County-Level Structural Racism and Birth Outcomes among Black and White Women. J Racial Ethn Health Disparities. 2018 Oct;5(5):966–77.
7. Eldridge L, Berrigan D. Structural Racism and Triple-Negative Breast Cancer Among Black and White Women in the United States. Health Equity. 2022;6(1):116–23.
8. Chantarat T, Van Riper DC, Hardeman RR. The intricacy of structural racism measurement: A pilot development of a latent-class multidimensional measure. EClinicalMedicine. 2021 Oct 1;40:101092.
9. O'Brien R, Neman T, Seltzer N, Evans L, Venkataramani A. Structural racism, economic opportunity and racial health disparities: Evidence from U.S. counties. SSM - Popul Health. 2020 Mar 9;11:100564.
10. Bowleg L, Malekzadeh AN, Mbaba M, Boone CA. Ending the HIV epidemic for all, not just some: structural racism as a fundamental but overlooked social-structural determinant of the US HIV epidemic. Curr Opin HIV AIDS. 2022 Mar 1;17(2):40–5.
11. Boynton-Jarrett R, Raj A, Inwards-Breland DJ. Structural integrity: Recognizing, measuring, and addressing systemic racism and its health impacts. EClinicalMedicine. 2021 Jun 3;36:100921.

12. Mendez DD, Hogan VK, Culhane JF. Institutional racism, neighborhood factors, stress, and preterm birth. Ethn Health. 2014;19(5):479–99.

13. Krivo LJ, Peterson RD, Kuhl DC. Segregation, racial structure, and neighborhood violent crime. AJS. 2009 May;114(6):1765–802.

14. Sewell AA. The Racism-Race Reification Process. Sociol Race Ethn. 2016;2(4):402.

15. Jacoby SF, Dong B, Beard JH, Wiebe DJ, Morrison CN. The enduring impact of historical and structural racism on urban violence in Philadelphia. Soc Sci Med 1982. 2018 Feb;199:87–95.

16. Houghton A, Jackson-Weaver O, Toraih E, Burley N, Byrne T, McGrew P, et al. Firearm homicide mortality is influenced by structural racism in US metropolitan areas. J Trauma Acute Care Surg. 2021 Jul 1;91(1):64–71.

17. Hardeman RR, Homan PA, Chantarat T, Davis BA, Brown TH. Improving The Measurement Of Structural Racism To Achieve Antiracist Health Policy. Health Aff (Millwood). 2022 Feb;41(2):179–86.

Supplement 2

SREI data sources

We relied on several additional data sources, in each case using the most recent data available, such as 2014 data from the U.S. Environmental Protection Agency's (EPA) National Air Toxin Assessment to measure total air toxins and cancer risk due to air pollutant exposure.(18) Variables about food availability based on distance to grocers for many populations were available from the Economic Research Service of the U.S. Department of Agriculture Food Access 2019 Research Atlas.(19) We accessed 2018 county-level data on the total jail population, pre-trial jail population, and prison population per 100,000 from the Vera Institute of Justice which aggregates local data and data from the U.S. Department of Justice Bureau of Justice Statistics Annual Survey of Jails and Census of Jails.(20) State and municipal law enforcement officer data from 2019 was provided by the Federal Bureau of Investigation Uniform Crime Reporting Program Police Employee Data for census places (municipalities) and states.(21) Per pupil spending data by school district from 2019 was obtained from the U.S. Census Bureau Annual Survey of School System Finances.(22) The U.S. Department of Housing and Urban Development and Department of Transportation Location Affordability Index Model Version 3 which uses 2012-2016 data was the source for job availability, retail job availability, and housing and transportation cost burdens for different household configurations.(23) Information on vacancy and foreclosure risk was obtained from U.S. Department of Housing and Urban Development Office of Policy Development and Research Neighborhood Stabilization program which uses 2010 data.(24) U.S. Census Bureau ACS Supplemental Poverty Measure resources provided an alternate poverty measure using 2019 data.(25) Local eviction data from 2016 was downloaded through the Princeton Eviction Lab. Eviction data for some census tracts are imputed based on data available at larger geographies.(26) The Healthcare Delivery Research Program National Cancer Institute provided a 2012 dataset on racial segregation including location quotients (LQ) and indices of concentration at the extremes (ICE) using measures described in Bemanian, et al.(27,28)

Comparison Indices, additional information

The ADI is available at the census block group level from the University of Wisconsin School of Medicine and Public Health Center for Health Disparities Research Neighborhood Atlas and is constructed from 17 ACS variables.(30,31) Organized into domains, the SVI is a composite of 16 ACS variables including demographic data such as ethnoracial group and disability status.(32) The SDI is a composite of seven ACS derived variables.(33) The COI creates scores from 29 variables organized into three domains. The variables include data from the ACS, the Department of Education, Environmental Protection Agency, and other Federal Agencies.

Variable selection

To select variables for inclusion, first, we used exploratory factor analysis to examine variables in each domain, clarifying some underlying patterns and leading to some changes to ensure that we measured what we intended. For example, the transportation domain originally seemed to be driven largely by urbanicity. Since transportation is important across all population densities, we included other variables that were less associated with urbanicity, such as carpooling. Second, we examined correlations and scatterplots within each domain to look for surprising directional, non-linear, or markedly high correlations between variables.

While examining many candidate variables, we sought fewer variables for the final index, valuing parsimony for several reasons. First, collinearity can obscure the impact of closely related variables in the same domain. Second, we sought to avoid creating a false sense of sophistication by including variables that would add little additional information (for example, “job availability” and “retail job availability” were 96% correlated, so only one was included). Finally, given our goal of developing a measure to be used in policy and advocacy, simplicity was a virtue.

The best approach to selecting an appropriate time frame for data was not initially clear. We opted for using the most recent data available at the time, both for the sake of simplicity and to best reflect current realities with shifting demographics, post-Covid emigration, urban revitalization efforts, etc. Though the ACS data used is 2015-2019 data, conceptually, the index captures more than just the status quo. For example, the education domain includes both current resources in the education system (per pupil spending) and the long-term effects of past resources in the education system (% of residents with a bachelor’s degree).

Where data were unavailable at the census tract level, we used one of two methods to determine a tract level value. For non-intersecting boundaries (county, state, etc.), the census tract adopted its “parent” geography’s value. For intersecting geographies (school district, ZCTA, etc.), we used weighted averages based on the fractions of the total land area in each census tract. Due to the irregular nature of some of the boundaries, such as school district, a population-weighted method is not immediately apparent as even census blocks are sometimes crossed.

Notes

18. Environmental Protection Agency, Office of Air Quality Planning and Standards. Air Toxics Screening Assessment, 2018 AirToxScreen Assessment Results [Internet] Research Triangle Park (NC): EPA; 2018 [cited 2022 Jun 21]. Available from: https://www.epa.gov/AirToxScreen/2018-airtoxscreen-assessment-results

19. US Department of Agriculture, Economic Research Service. Food Access Research Atlas [Internet]. Washington (DC): USDA ERS; 2021 Apr [cited 2022 Jun 21]. Available from: https://www.ers.usda.gov/data-products/food-access-research-atlas/

20. Vera Institute of Justice. Incarceration Trends Project [Internet]. Brooklyn, NY: Vera Institute of Justice; 2016 [cited 2022 Jun 21]. Available from: https://trends.vera.org

21. U.S. Department of Justice, Federal Bureau of Investigation, Criminal Justice Information Services Division, Full-time Law Enforcement Employees, by State by City (Table 78), 2019.; 2020 Oct. (Uniform Crime Report: Crime in the United States, 2019).

22. U.S. Census Bureau. Annual Survey of School System Finances, FY19 [Internet]. Washington (DC): U.S. Census Bureau; 2021 Apr [cited 2021 Dec 8]. Available from: https://www.census.gov/programs-surveys/school-finances.html

23. U.S. Department of Housing and Urban Development and Department of Transportation; Location Affordability Index [Internet]. Washington (DC): HUD and DOT; 2019 [cited

2021 Dec 15]. Available from: https://www.hudexchange.info/programs/location-affordability-index/

24. U.S. Department of Housing and Urban Development, Office of Policy Development and Research. HUD Neighborhood Stabilization Program [Internet]. Washington (DC): HUD; 2010 [cited 2021 Dec 18]. Available from: https://www.huduser.gov/portal/datasets/NSP.html

25. Fox L, Glassman B, Pacas J. The Supplemental Poverty Measure using the American Community Survey [Internet]. Washington (DC): US Census Bureau; 2020 Sep [cited 2022 Jun 21]. Available from: https://www.census.gov/content/dam/Census/library/working-papers/2020/demo/SEHSD-WP2020-09.html

26. Desmond M, Edmonds L, Hendrickson J, Krywokulski K, Leung L, Porton A. Eviction Lab National Database: Version 1.0 [Internet]. Princeton (NJ): Princeton University; 2018 [cited 2021 Oct 21]. Available from: www.evictionlab.org

27. National Cancer Institute, Healthcare Delivery Research Program. Social Determinants of Health by Census Tract [Internet]. Bethesda (MD): NCI HDRP; 2021 [cited 2021 Nov 21]. Created by Information Management Services, Inc. under US Government contracts HHSN261201500003B/75N91020F00001 to facilitate research activities of the NCI-funded Population-based Research to Optimize the Screening Process (PROSPR) consortium. Available from: https://healthcaredelivery.cancer.gov/social-determinants/

28. Bemanian A, Beyer KMM. Measures Matter: The Local Exposure/Isolation (LEx/Is) Metrics and Relationships between Local-Level Segregation and Breast Cancer Survival. Cancer Epidemiol Biomarkers Prev. 2017 Apr;26(4):516–24.

29. Kind AJH, Buckingham WR. Making Neighborhood-Disadvantage Metrics Accessible — The Neighborhood Atlas. N Engl J Med. 2018 Jun 28;378(26):2456–8.

30. University of Wisconsin School of Medicine and Public Health. 2019 Area Deprivation Index v3.1 [Internet]. 2021; [cited 2022 Apr 15]. Available from: https://www.neighborhoodatlas.medicine.wisc.edu/

31. Flanagan BE, Hallisey EJ, Adams E, Lavery A. Measuring Community Vulnerability to Natural and Anthropogenic Hazards: The Centers for Disease Control and Prevention's Social Vulnerability Index. 80(10):3.

32. Trinidad S, Brokamp C, Mor Huertas A, Beck AF, Riley CL, Rasnik E, et al. Use Of Area-Based Socioeconomic Deprivation Indices: A Scoping Review And Qualitative Analysis. Health Aff (Millwood). 2022 Dec;41(12):1804–11.

Supplement 3. Census tracts included in SREI construction and extent of imputation

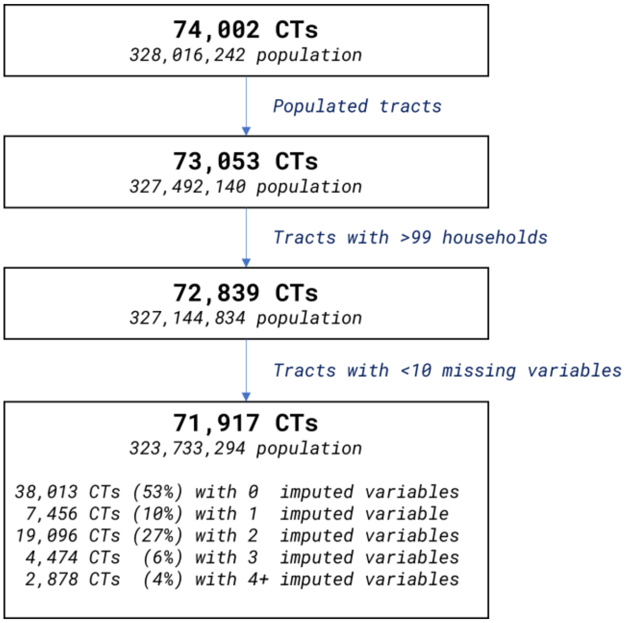

Supplement 4. Mean Life Expectancy, Diabetes Prevalence, and Ethnoracial Makeup by Decile of the Structural Racism Effect Index and Domains

Deciles	D1	D10	D1	D10	D1	D10
	**Outcomes**					
	Life Expectancy (years)		% Reporting Diabetes		% People of Color	
	85.0	70.9	6%	19%	3%	93%
**Indices**						
SREI	82.6	73.0	8%	17%	15%	69%
Wealth	82.6	72.9	8%	16%	20%	59%
Income & Poverty	82.5	73.9	9%	14%	14%	75%
Social Cohesion	81.4	73.3	8%	15%	10%	75%
Employment	81.9	73.8	8%	16%	22%	64%
Built Environment	81.7	73.9	9%	14%	16%	51%
Housing	81.4	74.5	8%	17%	14%	63%
Education	82.2	75.5	9%	16%	18%	68%
Transportation	80.0	75.3	9%	15%	38%	43%
Criminal Justice	79.9	75.7	8%	16%	23%	38%

Sources: Mean life expectancy from National Center for Health Statistics' US Small Area Life Expectancy Estimates Project (2010-2015). Diabetes Prevalence from Centers for Disease Control and Prevention PLACES Project (2020). Ethnoracial makeup from U.S. Census Bureau American Community Survey 2015-2019 estimates.

Notes: People of color is defined as Black, Latine, and Indigenous. ADI is Area Deprivation Index, SVI is Social Vulnerability Index, COI is Child Opportunity Index, SDI is Social Deprivation index, SREI is Structural Racism Effect Index. Unit of analysis is census tract. The top row of data shows the mean of each outcome by decile (i.e. the mean life expectancy in the decile of neighborhoods with the lowest life expectancy). The following rows show the mean value of the outcome per decile of neighborhoods ranked by the SREI and its individual domains (i.e. the average life expectancy in the decile of neighborhoods scoring the lowest on the index is 82.6 years).

Supplement 5. Structural Racism Effect Index Scores by U.S. Census Tract

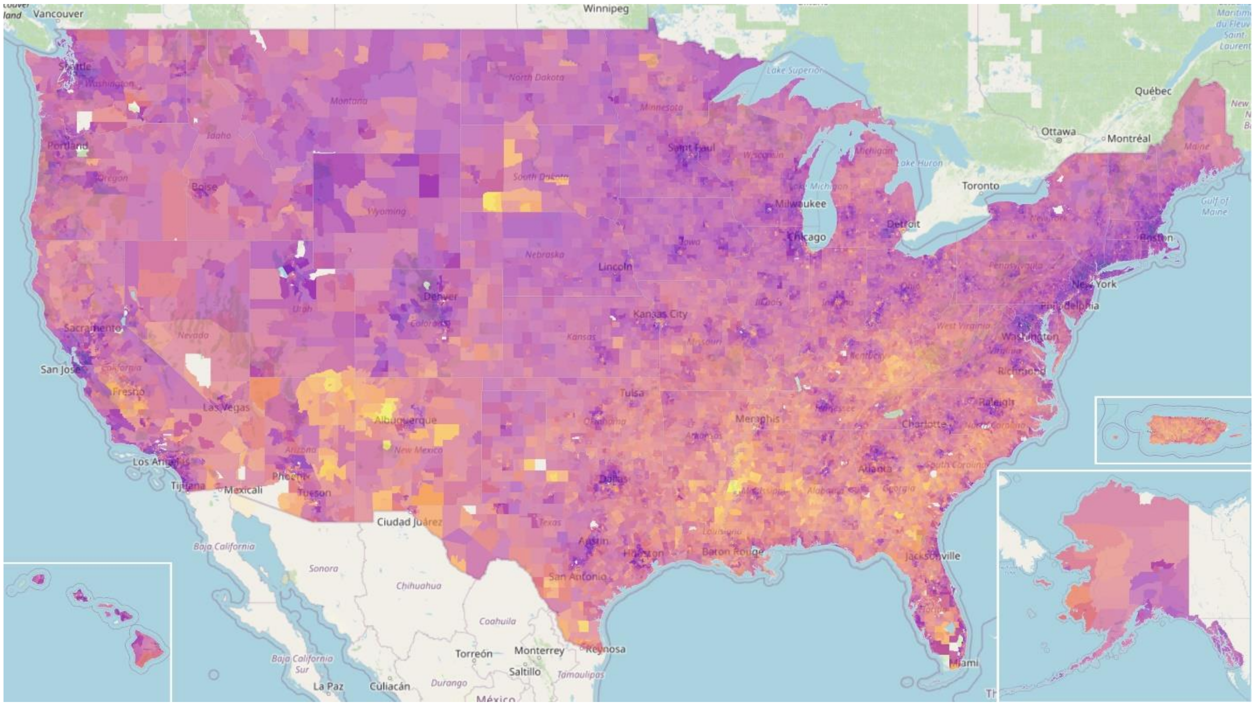

For each census tract, we mapped the SREI score and domain scores using ArcGis Pro Version 2.9.1. The map colors census tracts on a gradient where resource-rich areas (with lower SREI scores) use darker colors, and resource-deprived areas (with higher SREI scores) use lighter colors. Less populated areas are more transparent, while densely populated areas are more richly colored. Areas with no score are left uncolored.

The highest scores (indicating greater resource deprivation) are concentrated in the southeast United States, the Appalachian region, and Puerto Rico. Large areas with high SREI scores in the Southwest and Midwest correspond to Indian reservation land. Most large metropolitan areas appear dark (darker corresponds to greater availability of resources) on the map with a light center, with their urban cores experiencing serious structural deficits while resources concentrate in suburbs and exurbs.
